# Sex-specific traditional and disease-related risk factors for incident heart failure in patients with rheumatoid arthritis: a registry-based cohort study

**DOI:** 10.1007/s10067-026-08078-y

**Published:** 2026-04-09

**Authors:** Vera Zietemann, Tatjana Rudi, Daniel Bestler, Peter Herzer, Uta Kiltz, Christian Kneitz, Yvette Meissner, Anja Strangfeld

**Affiliations:** 1 Epidemiology and Health Services Research, German Rheumatology Research Center, Charitéplatz 1, 10117 Berlin, Germany; 2 Erfurt, Germany; 3 Scientific Advisory Board, RABBIT, Berlin, Germany; 4 Rheumazentrum Ruhrgebiet, Ruhr-Universität Bochum, Herne, Germany; 5 Schwerin, Germany; 6 Institute for Social Medicine, Epidemiology and Health Economics, Charité University Medicine, Berlin, Germany; 7 Department of Rheumatology and Clinical Immunology, Charité University Medicine, Berlin, Germany

**Keywords:** Comorbidity, Heart failure, Rheumatoid arthritis, Risk factors, Sex differences

## Abstract

**Objectives:**

To investigate associations with incident heart failure (HF) in patients with rheumatoid arthritis (RA) in relation to sex, traditional risk factors, and RA-specific factors, with analyses stratified by sex.

**Method:**

Patients without prevalent HF enrolled between January 2007 and June 2022 in the biologics register RABBIT were included and followed up to 10 years until December 2022. Patients were observed from the time of enrollment until the diagnosis of HF (outcome of interest), death, dropout, or end of study, whichever occurred first. The association of sex with HF and the association of traditional and RA-specific variables with HF, stratified by sex, were analyzed using multiple logistic regression.

**Results:**

The study sample consisted of 4022 men (3.9% HF) and 11,785 women (2.6% HF). For men compared to women, adjusted odds ratio for HF [95% CI] was 1.48 [1.20–1.83] overall, 2.42 [1.60–3.66] in patients with coronary heart disease (CHD), and 1.40 [1.10–1.78] for patients without CHD. Age, CHD, and high disease activity (DAS28-ESR) were the main risk factors for both sexes. The effect estimate of hypertension and particularly diabetes was higher in women than in men. Our results suggest a possible underdiagnosis of HF in women, especially of higher age.

**Conclusions:**

Our exploratory study suggests a higher chance for HF, particularly in men with CHD compared to women, among patients with RA. Age, CHD, and high disease activity may be important risk factors for HF for both sexes, whereas diabetes, hypertension, and longer RA duration seem to be more deleterious in women than men.

**Table:** 

**Key Points** •*Male RA patients with coronary heart disease (CHD) may have a higher chance for HF compared to females.* •*Effect estimates for diabetes and hypertension were higher in women than in men.* •*Age, CHD, and high RA disease activity were associated with HF in both sexes.*

**Supplementary Information:**

The online version contains supplementary material available at 10.1007/s10067-026-08078-y.

## Introduction

Heart failure (HF) is a complex clinical syndrome with different etiologies and phenotypes. HF occurring in the presence of coronary heart disease (CHD) is referred to as ischemic HF, which is the leading etiology (around 70%) in the general population [1, 2]. Heart abnormalities occurring without CHD are referred to as non-ischemic HF, with cardiomyopathies being the most common cause [3]. Patients with CHD are more likely to have HF with reduced ejection fraction (HFrEF), whereas HF with preserved ejection fraction (HFpEF) is thought to result mainly from non-ischemic etiologies [2, 4, 5]. The pathophysiology of HFpEF is complex and heterogeneous. Important components are microvascular dysfunction due to longstanding high systemic inflammation and impaired myocardial perfusion [2, 4–6]. Rheumatoid arthritis (RA) is an immune-mediated chronic inflammatory disease, associated with an almost twofold increased risk of HF compared to the general population [5, 7–9]. Elevated systemic inflammation is associated with an increased risk for HFpEF and non-ischemic HF [10–12]. In the RA population, non-ischemic HF seems to be at least as common as ischemic HF [3, 5, 13].

Sex-specific differences in HF phenotypes, with HFrEF predominating in men and HFpEF in older women, were identified in the general population [1, 6, 14]. Furthermore, differences in HF risk between women and men were described with a higher impact of diabetes, hypertension, obesity, smoking, systolic blood pressure, heart rate, C-reactive protein (CRP), and N-terminal pro-B-type natriuretic peptide for women than men [15, 16]. To our knowledge, no study has explicitly examined sex-specific risk factors for HF in patients with RA. Crowson et al. found a higher risk of cardiovascular disease (CVD) in men compared to women [17]. Age, smoking, and hypertension were significant risk factors for both sexes, whereas higher triglycerides were only significant in men and diabetes only in women. Disease activity measures of RA such as the erythrocyte sedimentation rate (ESR), the composite score DAS28-ESR, as well as the presence of rheumatoid factor (RF) and/or anti-citrullinated protein antibodies (ACPA) were not significantly associated with CVD risk after adjustment for traditional risk factors [17]. However, RA-specific variables seemed to have a greater impact among women than men [17].

The evaluation of sex-specific risk factors for CVD is not without pitfalls. For the general population, a diagnosis gap was shown with fewer women being diagnosed but more dying from CVD [18, 19]. Cardiac diseases in women are often overlooked during routine screening. Women are typically diagnosed later and present with more severe symptoms at onset than men [15, 20, 21]. In patients with RA, HF may be underdiagnosed as its symptoms are difficult to ascertain due to atypical presentations and concomitant diseases [12, 13, 22]. Symptoms of chronic HF may be wrongly attributed to RA disease activity (e.g., fatigue), and HF may therefore be diagnosed with lag time [23]. Ferreira et al. showed that 32% of patients with RA fulfilled the criteria for HF, but only 6.8% were diagnosed [24]. In RA patients, cardiovascular risk was found to be especially underestimated in women [25].

Therefore, the objectives of our exploratory, register-based cohort study were (I) to investigate the association between sex and incident HF and (II) to investigate the association between traditional and RA-specific factors and HF incidence, stratified by sex. Furthermore, the possibility of an underdiagnosis of HF was visually explored.

## Materials and methods

### Data source

The German biologics register RABBIT (Rheumatoid Arthritis: Observation of Biologic Therapy) is an ongoing, prospective longitudinally followed cohort of adult patients with RA with age at disease onset > 15 years. Patients are included with a new start of a biologic or targeted synthetic disease-modifying anti-rheumatic drug (DMARD), or with a conventional synthetic DMARD (csDMARD) after at least one prior DMARD therapy. At the time of enrollment, at months 3 and 6, and then every 6 months during the observation period, information is collected from patients and rheumatologists on demographics, clinical status, DMARD treatment regimen, concomitant therapies with glucocorticoids (GC) and non-steroidal anti-inflammatory drugs (NSAID), including cyclooxygenase-II (Cox-II)-inhibitors, at every follow-up visit. Adverse events are reported from treating rheumatologists and classified as serious or non-serious according to the International Conference on Harmonization E2A guidelines [26]. Every adverse event is coded in the study center according to the Medical Dictionary for Regulatory Activities (MedDRA) [27]. Comorbidities are reported at enrollment and every 2.5 years after enrollment. Details of the RABBIT register are described elsewhere [28]. The study protocol was approved by the ethics committee of the Charité University Medicine Berlin, Germany (EA4/123/21). Before enrollment, patients gave written informed consent.

### Patient population

**Fig. 1:**
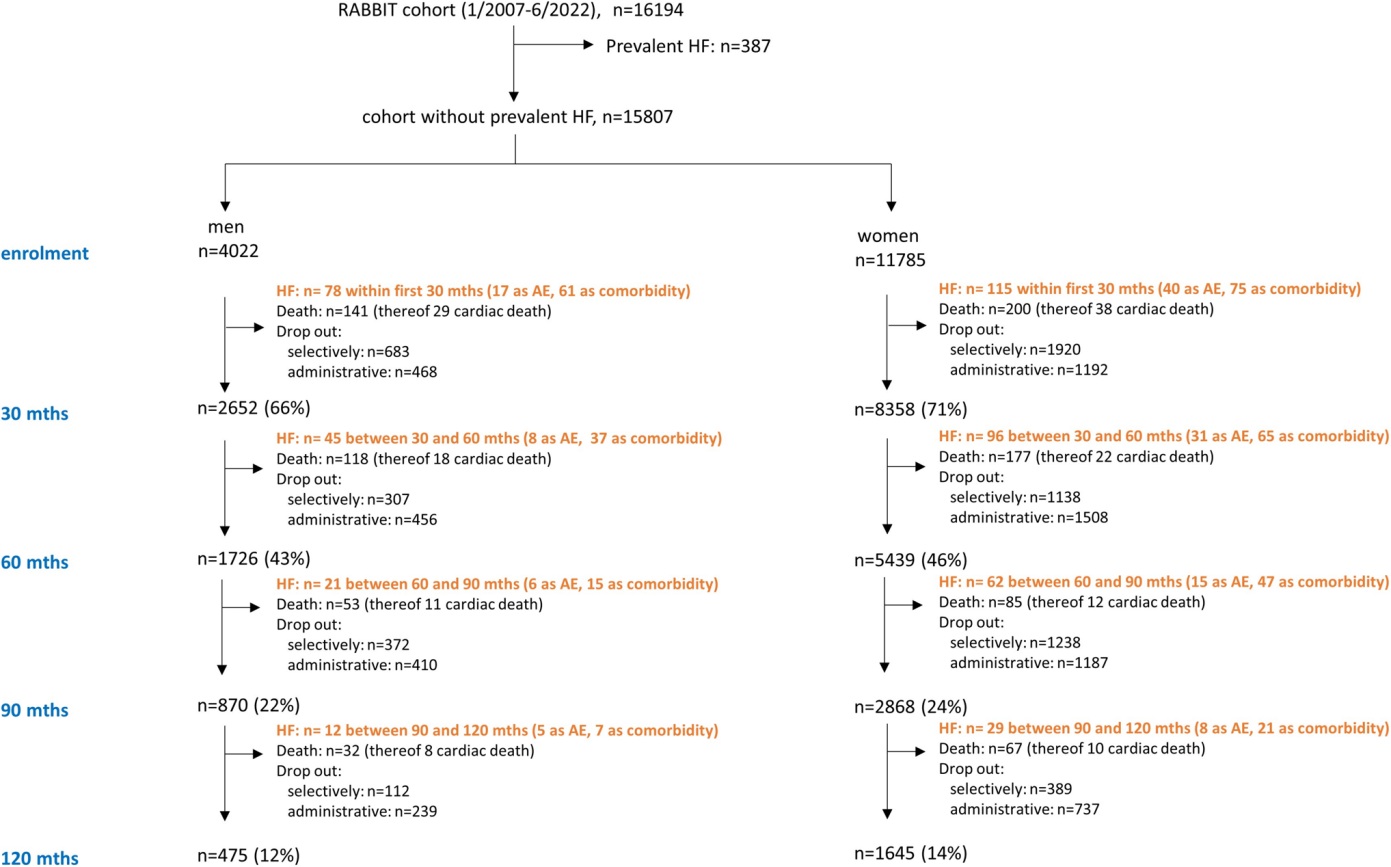
Flow chart stratified by sex. AE, adverse event; HF, heart failure; mths, months

Patients with RA enrolled between January 2007 and June 2022 without reported prevalent HF were included in the analysis (Fig. 1). The end of observation time was December 2022.

### Definition of outcome

The primary outcome was defined as the first report of HF by the rheumatologist. HF events were coded as one of the following MedDRA codes on preferred-term level were selected: acute left ventricular failure, cardiac failure, cardiac failure acute, cardiac failure chronic, cardiac failure congestive, left ventricular failure and right ventricular failure. If available, hospital discharge letters and death certificates were reviewed. In addition to the adverse event reporting, the presence of HF as a comorbid condition is regularly asked for and captured every 2.5 years after enrollment. The first report of HF—either as an adverse event or as a comorbid condition—was counted as an incident event. As the information about ischemic and non-ischemic HF is not available in the register, CHD history was applied as a proxy [29]. We defined ischemic HF as having a documented history of CHD prior to HF, and non-ischemic HF as having no documented CHD history. Severity of HF was classified based on the New York Heart Association (NYHA) criteria.

As a secondary outcome, the first reporting of incidents of HF or cardiac death was used as a composite outcome (see Supplemental Methods for MedDRA codes).

### Definition of sex and possible risk factors for the outcome

Sex was used as a binary variable (men versus women). This information was based on the documentation from the patient and the rheumatologist. Our approach did not capture the complex interplay between sex and gender, as this was not the focus of our research.

Possible risk factors were documented by the rheumatologist and determined a priori based on existing literature. We analyzed variables to cover traditional cardiovascular [15, 17, 22, 30] and RA-specific risk factors [22]. Traditional factors included age, ever diagnosed CHD, hypertension, diabetes, hyperlipidemia, obesity (defined as BMI ≥ 30 kg/m^2^) and ever smoking (former and current smoking). Factors associated with RA included disease activity assessed by DAS28-ESR, CRP (≥ 5 mg/l versus < 5 mg/l), RA disease duration, RF and/or ACPA seropositive, GC treatment during the 6 months prior to baseline (< 5 mg/day, 5–10 mg/day, ≥ 10 mg/day), and concomitant NSAID and/or COX-II-inhibitor use.

### Study design

The objective of this study was to identify sex-specific risk factors from the list above associated with the development of HF within a clinically relevant observation window, i.e., assessing which patient characteristics are linked to an increased probability of developing HF. Baseline was defined as the time of enrollment into RABBIT. Patients were observed until the occurrence of the outcome, dropout, death or end of study, whichever occurred first. Patients could have been observed for up to 10 years, and therefore, the probability of the outcome occurring within 10 years was investigated (main analysis).

As additional analysis, we restricted the observation period of each patient to a maximum of 2.5 years preceding the outcome for patients with incident HF, or prior to dropout, death, or end of study for all other patients. Baseline was individually defined for every patient depending on the outcome occurrence or his/her duration of follow-up. Therefore, baseline could have been either at enrollment, at months 30, 60, or 90. In this additional analysis, the probability of the outcome occurring within 2.5 years was investigated (Supplemental Methods and Supplemental Fig.1).

### Statistical analysis

Patient characteristics at baseline stratified by sex are presented using descriptive statistics (numbers, percentages, mean, and standard deviation). The number of missing values is given in Supplemental Table 1.

**Table 1:** Baseline characteristics stratified by sex using enrollment into RABBIT as baseline

	Men (*n* = 4022)	Women (*n* = 11,785)
**Demographics and clinical information**		
Age (years)	58.2 ± 11.8	57.3 ± 13.1
Age < 50 years	859 (21.4)	2977 (25.3)
RA disease duration (years)	7.4 ± 7.6	9.8 ± 9.1
RF and/or ACPA seropositive	2939 (77.0)	8542 (76.7)
Joint erosions	2111 (55.3)	5906 (52.8)
RA disease activity (DAS28-ESR)	4.7 ± 1.4	4.8 ± 1.3
ESR (mm/h)	28.6 ± 23.7	26.9 ± 21.2
ESR > 21 mm/h	1836 (49.7)	5300 (48.7)
CRP (mg/l)	16.9 ± 23.8	12.3 ± 17.8
CRP ≥ 5 mg/l	2318 (63.8)	5911 (55.6)
Swollen joint count [0–28]	5.1 ± 4.9	5.0 ± 4.6
Tender joint count [0–28]	7.2 ± 6.4	7.6 ± 6.4
% of full physical function (FFbH) [0–100]	71.9 ± 21.9	65.1 ± 23.1
Patient reported global health [0–10]	5.6 ± 2.1	5.7 ± 2.1
Patient reported fatigue [0–10]	4.7 ± 2.7	5.3 ± 2.7
Patient reported pain [0–10]	5.6 ± 2.3	5.8 ± 2.3
**Previous treatments**		
*N* of previous csDMARDs	1.2 ± 1.0	1.5 ± 1.0
*N* of previous bDMARDs	0.3 ± 0.6	0.4 ± 0.6
*N* of previous tsDMARDs	0 ± 0.1	0 ± 0.1
GC dosage 6 months prior to baseline		
< 5 mg/d	1900 (48.4)	5876 (51.3)
5 to < 10 mg/d	1324 (33.7)	4144 (36.2)
≥ 10 mg/d	702 (17.9)	1427 (12.5)
**Current DMARD treatment**		
Tumor necrosis factor inhibitor	1712 (42.6)	4835 (41.0)
Interleukin-6 inhibitor	341 (8.5)	1132 (9.6)
Abatacept	217 (5.4)	654 (5.5)
Rituximab	320 (8.0)	1078 (9.1)
Janus kinase inhibitor	296 (7.4)	890 (7.5)
csDMARD	1128 (28.0)	3166 (26.9)
**Concomitant medication**		
b/tsDMARD combined with csDMARD	1960 (67.9)	5168 (60.2)
Oral GC	3173 (80.6)	8685 (75.7)
NSAID or Cox-II inhibitor	1583 (39.4)	5045 (42.8)
**History of comorbidities**		
*N* of comorbidities*	1.1 ± 1.2	1.0 ± 1.1
Hypertension	1740 (43.3)	4844 (41.1)
Coronary heart disease	441 (11.0)	516 (4.4)
History of stroke/transient ischemic attack	101 (2.5)	178 (1.5)
Hyperlipidemia	416 (10.3)	1006 (8.5)
Diabetes	555 (13.8)	1232 (10.4)
Chronic obstructive pulmonary disease/lung fibrosis	305 (7.6)	571 (4.8)
Chronic renal disease	193 (4.8)	542 (4.6)
Cancer	186 (4.6)	475 (4.0)
Osteoporosis	375 (9.3)	2029 (17.2)
Obesity (body mass index ≥ 30 kg/m^2^)	1027 (25.9)	3168 (27.2)
Smoking, ever	3045 (76.3)	5844 (50.1)

Values are numbers (percent) or means ± standard deviation

*ACPA* anti-citrullinated protein antibodies, *bDMARD* biologic disease-modifying anti-rheumatic drug, *CRP* C-reactive protein, *csDMARD* conventional synthetic disease-modifying anti-rheumatic drug, *DAS28-ESR* disease activity score in 28 joints, *ESR* erythrocyte sedimentation rate, *FFbH* Hannover functional status questionnaire, *GC* glucocorticoid, *NSAID* non-steroidal anti-inflammatory drug, *RF* rheumatoid factor, *tsDMARD* targeted synthetic disease-modifying anti-rheumatic drug

^*^N of comorbidities comprises sum of: hypertension, coronary heart disease, stroke/transient ischemic attack, hyperlipidemia, diabetes, chronic obstructive pulmonary disease/lung fibrosis, chronic renal disease, cancer, and osteoporosis

To investigate the association between sex and HF (Aim I), sex was used as the exposure of interest. The association between sex and outcome was analyzed using multiple logistic regression adjusted for age (Model 1) and for traditional and RA-specific risk factors listed above (Model 2). For Aim II, the association between individual risk factors and outcome was examined using multiple logistic regression, applied separately for men and women. To investigate if the effect of a factor on the outcome was different for men compared to women due to effect modification, each risk factor was tested for interaction with sex using the likelihood ratio test.

For both aims, linearity was checked for continuous variables using restricted cubic splines with 5 knots and Wald test statistic [31]. To adjust for selective dropout, we used inverse probability of censoring weighting [32]. Obtained weights were bounded at the 1 st and 99th percentile to reduce the influence of extreme values [33]. Selective dropout was defined as any dropout except study termination due to study end (next visit lies in the future, administrative censoring). In addition, any death was counted as selective dropout in the main analysis. For the analysis of the composite outcome, all deaths other than from cardiac causes were counted as selective dropouts. Possible underdiagnosis of HF was visualized by plotting the odds ratio (OR) against age for the outcome HF and the composite outcome HF or cardiac death using restricted cubic splines with 5 knots [31]. Single imputation was performed by regression with fully conditional specification, separately for the main and the additional analysis. *P*-values in regression models < 0.05 were considered statistically significant. Data analyses were performed with SAS V.9.4 (SAS Institute, Cary NC).

### Patient and public involvement

Results were presented and discussed in the presence of patient research partners of the TARISMA (Targeted Risk Management in Musculoskeletal Diseases) research network.

## Results

Out of 16,194 RA patients, 387 were excluded due to reporting of HF at enrollment (prevalent HF cases, 35% men) (Fig. 1). Women with prevalent HF were around 2 years older than men (women, 69.9 years; men, 67.7 years) and had an almost 6 years longer RA disease duration (women, 13.8 years; men, 7.9 years).

The study cohort without prevalent HF consisted of 4022 men and 11,785 women. Figure 1 shows numbers of HF events, deaths, and dropouts for every period of 30 months, stratified by sex. Median observation time was 49.7 months (quartile 1–3, 22.7–83.6 months) in men and 54.0 months (quartile 1–3, 23.9–89.1 months) in women.

Patient characteristics at baseline stratified by sex are given in Table 1. Compared to women, men were slightly older, had a shorter RA disease duration, similar disease activity parameters, but higher CRP values, especially more frequently CRP ≥ 5 mg/l. In contrast, women presented with lower physical function and higher fatigue and pain levels. Treatment with DMARDs was comparable between sexes. However, men were more likely to receive concomitant treatment with csDMARDs and GC, while women were more likely to receive treatment with NSAIDs and/or Cox-II-inhibitors. Traditional CVD risk factors such as CHD, hyperlipidemia, diabetes and smoking were more frequently present in men, but women showed a higher prevalence of osteoporosis. Patient characteristics were similar using baseline up to 2.5 years prior to HF (Supplemental Table 2).

### Aim (I): association between sex and outcome

Incident HF was reported for 156 men (3.9%) and 302 women (2.6%). Cardiac death was observed in 80 men (2.0%) and 110 women (0.9%), and the composite outcome of HF or cardiac death (secondary outcome) was reported for 222 men (5.5%) and 384 women (3.3%).

**Fig. 2:**
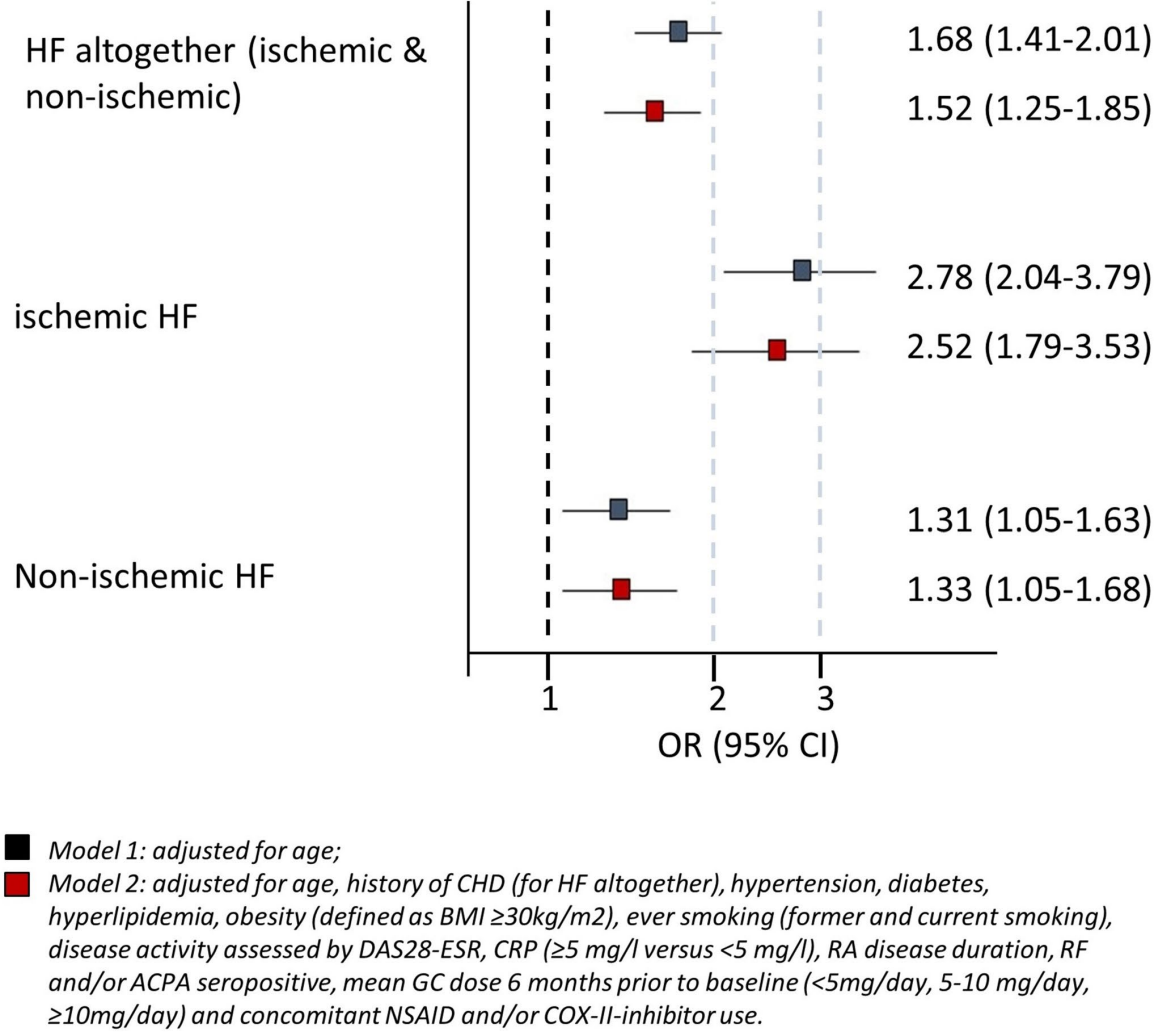
Association between sex and heart failure, with females used as reference. ACPA, anti-citrullinated protein antibodies; BMI, body mass index; CHD, coronary heart disease; CI, confidence interval; Cox-II inhibitors, inhibitors of cyclooxygenase-II; CRP, C-reactive protein; GC, glucocorticoid; HF, heart failure; NSAID, non-steroidal anti-inflammatory drug; OR, odds ratio; RA, rheumatoid arthritis; RF, rheumatoid factor

Male sex was significantly associated with incident HF, regardless of adjustment (Fig. 2). A more than twofold increased odds of HF for men compared to women was found for ischemic HF (66 men, 78 women). The difference between women and men was lower for non-ischemic HF (90 men, 224 women) but remained significant.

Results were similar in the additional analysis (Supplemental Table 3). Furthermore, men had a higher chance for the secondary outcome (Supplemental Table 4).

### Aim (II): association between possible risk factors and outcome, stratified by sex

**Fig. 3:**
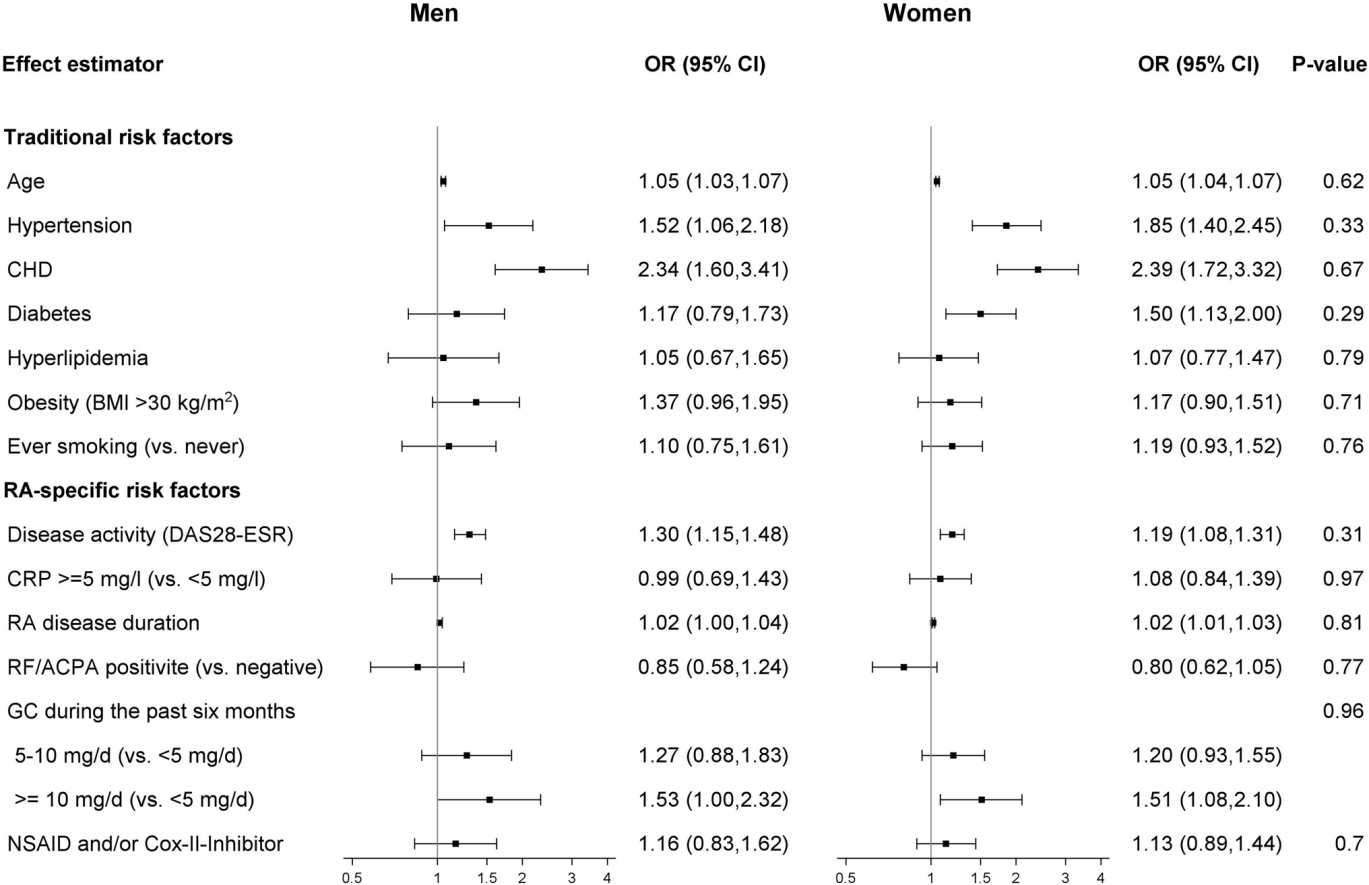
Odds ratio of each predictor in a fully adjusted model stratified by sex for the outcome of heart failure within 10 years of follow-up. All analyses are adjusted for selective drop out using inverse probability of censoring weighting. *P*-value, *p*-value of the interaction term; ACPA, anti-citrullinated protein antibodies; BMI, body mass index; CHD, coronary heart disease; CI, confidence interval; Cox-II inhibitors, inhibitors of cyclooxygenase-II; CRP, C-reactive protein; DAS28-ESR, disease activity score in 28 joints; ESR, erythrocyte sedimentation rate; GC, glucocorticoids; NSAID, non-steroidal anti-inflammatory drugs; OR, odds ratio; RA, rheumatoid arthritis; RF, rheumatoid factor

Baseline characteristics that were significantly associated with HF in both sexes comprised the traditional risk factors age, hypertension, CHD, and the RA-specific factors disease activity and longer RA duration (Fig. 3; for *p*-values, see Supplemental Table 5). Furthermore, a GC dosage ≥ 10 mg/d was significantly associated with HI in both sexes. The OR for diabetes was 1.50 (95% CI, 1.13–2.00) in women, but only 1.17 (95% CI, 0.79–1.73) in men; however, there was no significant interaction term.

Results of the additional analysis were quite similar to the main results (Supplemental Fig. 2, Supplemental Table 5). However, a significant association between CRP-values ≥ 5 mg/l and HF was found but only in women.

Regarding the secondary outcome, results of the main analysis were comparable to those of the main analysis using the primary outcome (Supplemental Fig. 3, Supplemental Table 5), with the addition that obesity showed a significant association in men and smoking in women. The additional analysis likewise found the association with obesity for men. In women, there was a significant association between higher CRP values and the composite outcome, as seen for the primary outcome. The interaction term for diabetes was significant using the composite outcome in the models (Supplemental Fig. 3).

### Visualization of the underdiagnosis of HF

**Fig. 4:**
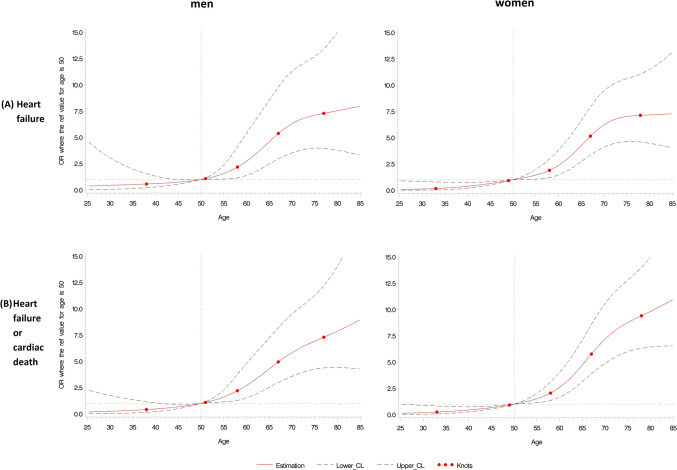
Visualization of the association between **A** age and HF and **B** age and the composite outcome (HF or cardiac death), applied separately for men and women

Figure 4 shows the association of age with the primary and secondary outcome stratified by sex. For women, the curve for HF and for the composite outcome is steep between 60 and 75 years and continues to rise afterward only for the composite outcome, i.e., a clear plateau can be recognized from the age of 75 years onwards for HF, whereas the curve does not show a plateau for the composite outcome HF or cardiac death. For men, there is only some evidence for a plateau for older ages for HF and there is no plateau for the composite outcome.

Classification of HF based on the NYHA criteria showed greater severity in women compared to men at the time of first HF documentation. If the HF was reported as an adverse event, 90% of cases in women and 86% in men were reported as severe. If the HF was reported as a comorbidity, 41% of women and 35% of men had HF with NYHA > 2. The NYHA criteria were not available for 37% of men and 42% of women.

## Discussion

This exploratory study investigated sex-related differences in the incidence of HF in more than 16,000 patients with RA observed in daily rheumatology practice. We found a higher prevalence of traditional CVD risk factors in men than in women with RA. The odds for the occurrence of an incident HF were significantly elevated in men compared to women, and this was mainly driven by a 2.4-fold higher chance for ischemic HF, whereas the difference for non-ischemic HF was much lower. Factors associated with the reporting of HF within the following 10 years were older age, the presence of CHD and hypertension, higher RA disease activity, longer RA duration and higher GC dosages in both sexes. The association between hypertension and HF was more pronounced in women than in men. A significant association between diabetes and HF was only found in women, not in men. Furthermore, higher CRP values were associated with HF in women when the time between documentation of CRP and outcome was limited to a maximum of 2.5 years. Beside this, our results suggest a possible underdiagnosis of HF especially in elderly women.

In the general population, men were found to have a higher risk of HF compared to women [16]. For patients with RA, only the risk of CVD was studied so far and a higher risk of CVD for male patients compared to female patients was found [17, 34]. In our study sample, the odds for the outcome were significantly higher for men compared to women in all models. In RA populations, sex differences in the prevalence of comorbidities, in disease activity indices and CRP levels have been reported [17, 35, 36]. In our cohort, men were more likely to have traditional risk factors, especially CHD, and the ischemic etiology of HF was more common in men than women, as it was previously reported for the general population and patients with RA [15, 20].

Risk calculators developed in the general population for cardiovascular risk failed to perform well in RA patients [37]. To our knowledge, our exploratory study is the first to investigate risk factors for HF stratified by sex for patients with RA. Age and CHD were the strongest traditional factors for both men and women. Comparable to the study by Crowson et al., which investigated CVD risk in patients with RA, diabetes was significantly associated with HF only in women in all our models [17]. As known for the general population [15, 38], we could show that hypertension and especially diabetes were more strongly associated with HF in females than in male patients with RA.

In contrast to Crowson et al. [17] and Raadsen et al. [39], who did not find an increased CVD risk for RA-specific factors, we found a significant association between RA disease activity (DAS28-ESR) and HF for both sexes. Currently it is thought that systemic inflammation has a greater impact in women than men with regard to the progression of HF [15, 38], and CRP is assumed to be a better predictor for the development of HF in women than in men in the general population [18, 40]. However, the causal relationship between CRP and HF risk is still under discussion [41], and an association between inflammatory markers and HF was especially found when using shorter follow-up times [10, 42]. In our exploratory study, a high CRP value was a significantly associated factor only in women and only if measured up to 2.5 years prior to HF reporting.

No clear association was seen for the remaining investigated factors from either the main or the additional analyses. Referring to the lipid paradox described in patients with RA [17, 30], we could not find an association for patients with hyperlipidemia. Obesity measured by BMI was only found to be a significant factor for men for the combined outcome of HF and/or cardiac death. However, patients with RA may have a higher percentage of fat and lower muscle mass (rheumatoid cachexia), and obesity measured by BMI may not capture the proposed effect of central obesity on HF risk especially in women, who have higher subcutaneous adipose tissue [15, 18, 30]. In contrast to studies on HF and CVD as outcomes in RA and non-RA populations [16, 17], we could not find a strong negative association of smoking with HF in our cohort based on patients with advanced RA. However, smoking is associated with an increased risk of RA and therefore prone to index event bias if analyzing the outcome HF in RA patients [43–45]. Therefore, conditioning on RA as an index event may explain why this traditional risk factor was not strongly associated with HF in our exploratory study.

In accordance with the study by Crowson et al. [17], we did not find a significant association of the combined exposure RF and/or ACPA positivity with our outcome. However, RF seems to have a higher impact on HF, CVD, and cardiovascular death, whereas ACPA seems to have a higher impact on ischemic heart disease [30]. Further studies need to investigate the association of autoantibodies with HF. We found an association for higher GC doses compared to low doses for men and women, but there was no clear dose dependency in our analyses. This may be due to the complex interplay between beneficial effects (lowering disease activity) and harmful effects (intrinsic toxic effects), resulting in conflicting evidence [30]. Likewise, no significant association could be seen for NSAID and/or Cox-II inhibitor use in our exploratory study.

Our results must be interpreted with caution due to the exploratory approach and the possibility of selection bias and underdiagnosis of HF, especially in women. As shown for the general population by Magnussen et al. [16], men developed HF earlier than women in middle-aged to older age groups, but women revealed a higher HF incidence in the oldest group (> 80 years). Some evidence of a plateau in the curve for HF incidence could only be seen for the very old (> 90 years of age) [16]. Our analysis provides an initial indication that especially in elderly women with RA, there may be an underdiagnosis of HF, as the plateau seen for HF for elderly women was less obvious using the combined outcome HF and/or cardiac death. HF symptoms, and especially the phenotype HFpEF, may be difficult to ascertain due to diseases like osteoporosis and functional limitation [13, 46], which are often found in elderly women with RA.

This is an exploratory, register-based cohort study investigating association; therefore, the effect of the investigated factors on the outcome should not be interpreted causally. The main limitations of our study were that the exact date for the HF diagnosis was only reported for 22% of the men and 31% of the women and that we did not have details on HF phenotypes and HF symptoms for most patients. This impeded application of time-to-event modeling strategies. In addition, the distinction between ischemic and non-ischemic HF was based solely on a history of CHD, as previously performed in another observational RA study from Sweden [29]. Furthermore, we do not have information on the severity of hypertension, diabetes, hyperlipidemia, and CHD; therefore, it was impossible to evaluate if men and women were adequately treated for these traditional risk factors. Unfortunately, data on waist and hip circumference are not available, and we did not have information about the diagnosis date of HF for patients with prevalent HF at enrollment into RABBIT. Therefore, elderly women with HF could be missing due to the study design, as enrollment did not start at RA diagnosis where HF risk may be increased [5]. In any case, rheumatologists need to be aware of the problem of underdiagnosis of cardiac disease in women if using routine exams [21]. As the results are based on a German registry, conclusions may not be generalizable to other healthcare settings and ethnic populations.

Our study has several strengths. To our knowledge, this is the first study investigating traditional and RA-specific risk factors for HF stratified by sex using a large cohort of patients with RA. In the main analysis, patients were observed up to 10 years as the progression of HF is gradual with subclinical and asymptomatic changes existing over many years [22] and HF may be unrecognized several years due to mechanisms to compensate for insufficient pump performance. However, we furthermore performed an additional analysis using a follow-up time up to 2.5 years to limit the period between risk factor documentation and outcome. Additionally, we analyzed the outcome HF and the composite outcome HF or cardiac death to take into account the possible underdiagnosis of HF in patients with RA. We also carefully accounted for selective dropouts.

## Conclusion

This is the first study to investigate sex-specific risk factors for HF in RA. Our exploratory study suggests a higher chance for HF in men compared to women among patients with RA. This association was mainly based on a more than twofold higher chance for ischemic HF. Traditional and RA-specific risk factors for HF were found for both women and men, with age, CHD, and RA disease activity being the most important variables. As described for the general population, diabetes, hypertension, and high CRP values were more strongly associated with HF in women than men with RA. Furthermore, our results suggest a possible underdiagnosis of HF especially in elderly women. As recommended by the EULAR guidelines [7], rheumatologists are responsible for CVD risk management, but implementation may still be insufficient [34]. Further sex-specific research is necessary to verify our results and to clarify if sex-specific guidelines for diagnosis of HF and therapies for HF risk factors may be needed for patients with RA, as discussed for the general population [15, 19].

## Supplementary Information

Below is the link to the electronic supplementary material.

ESM 1 (PDF 1.13 MB)

## Data Availability

In the RABBIT study, patients’ consent does not include approval to share their data. Data therefore cannot be made available.

## Declarations

### Patient and public involvement

Results were presented and discussed in the presence of patient research partners of the TARISMA (Targeted Risk Management in Musculoskeletal Diseases) research network.
