# Supplementary material for: Sex-specific traditional and disease-related risk factors for incident heart failure in patients with rheumatoid arthritis: a registry-based cohort study
Source: Clin Rheumatol. 2026 Apr 9;45(6):3143–54. doi: 10.1007/s10067-026-08078-y (PMC13249704; doi:10.1007/s10067-026-08078-y)
Supplement: Supplementary file 1 — (PDF 1.13 MB) [file 10067_2026_8078_MOESM1_ESM.pdf]

Sex-specific traditional and rheumatoid arthritis specific risk factors for incident heart failure in patients with rheumatoid arthritis: a registry-based cohort study

Supplementary material

Clinical Rheumatology

Vera Zietemann[1], Tatjana Rudi[1], Daniel Bestler[2], Peter Herzer[3], Uta Kiltz[4], Christian Kneitz[5], Yvette Meissner[1,6], Anja Strangfeld[1,7]

1 Epidemiology and Health Services Research, German Rheumatology Research Center, Berlin, Germany

2 Rheumatologist, Private Practice, Erfurt, Germany

3 Scientific Advisory Board, RABBIT, Berlin, Germany

4 Ruhr-Universität Bochum, and Rheumazentrum Ruhrgebiet, Herne, Germany

5 Rheumatologist, Private Practice, Schwerin, Germany

6 Institute for Social Medicine, Epidemiology and Health Economics, Charité University Medicine, Berlin, Germany

7 Department of Rheumatology and Clinical Immunology, Charité University Medicine, Berlin, Germany

Correspondence to

Yvette Meissner,

German Rheumatology Research Center, Programme Area Epidemiology and Health Services Research, Charitéplatz 1, 10117 Berlin, Germany; Phone: +49 30 28460 623; E-Mail: y.meissner@drfz.de

SUPPLEMENTAL METHODS

Alternative analysis: definition outcome

Composite outcome of incident HF and/or cardiac death

The following MedDRA codes were used for cardiac death (number of patients):

myocardial infarction (55), cardiac failure (49), sudden cardiac death (22), cardiac arrest (13), acute myocardial infarction (12), cardiac failure congestive (11), left ventricular failure (11), right ventricular failure (7), cardiac failure acute (6), cardiogenic shock (6), arrhythmia (5), cardiac death (3), cardiopulmonary failure (3), ischemic cardiomyopathy (3), ventricular fibrillation (3), atrial fibrillation (2), cardiovascular insufficiency (2), coronary artery disease (2), pulmonary edema (2), tachyarrhythmia (2), acute coronary syndrome (1), acute left ventricular failure (1), acute right ventricular failure (1), aortic valve stenosis (1), atrioventricular block complete (1), cardiac disorder (1), cardiac failure chronic (1), cardiac tamonade (1), cardio-respiratory arrest (1), congestive cardiomyopathy (1), Cor pulmonale (1), low cardiac output syndrome (1), myocarditis (1), and pulseless electrical activity (1)

Alternative analysis: definition of the study design

Supplemental figure 1 illustrates the study design.

For the main analysis, patient characteristics at enrolment into RABBIT (orange rectangle) were used as predictors for the outcome within up to 10 years, presented as orange lines.

For the additional analysis, the follow-up time of each patient was limited to 2.5 year observation period and baseline was defined according to the period in which the outcome occurred (drop out, death, or end of study). Eligible baseline time points were pre-defined according to the study design of RABBIT with comorbid condition capturing every 30 months. For example considering patients 4 and 5, baseline was defined as the time of enrolment as follow-up for these patients was only up to 30 months (patient 4 was censored and patient 5 had an event). For patients 2 and 8, baseline was defined at month 30 as follow-up was up to month 60 (patient 2 had an event and patient 8 was censored). For patients 6 and 7, baseline was defined at month 60 as follow-up was up to month 90 (patients 6 was censored and patient 7 had an event). For patients 1 and 3, baseline was defined at month 90 as follow-up was up to month 120 (patient 1 had an event and patient 3 was censored). Therefore, each patient is represented only once. The probability of the outcome occurring within 2.5 years was investigated.

Supplemental Figure 1. Study design exemplifying 8 patients with and without outcomes to illustrate the definition of baseline (marked as a rectangle) for the main analysis (up to 10 years of follow-up, shown in orange) and the additional analysis (up to 2.5 years of follow-up, shown in blue)

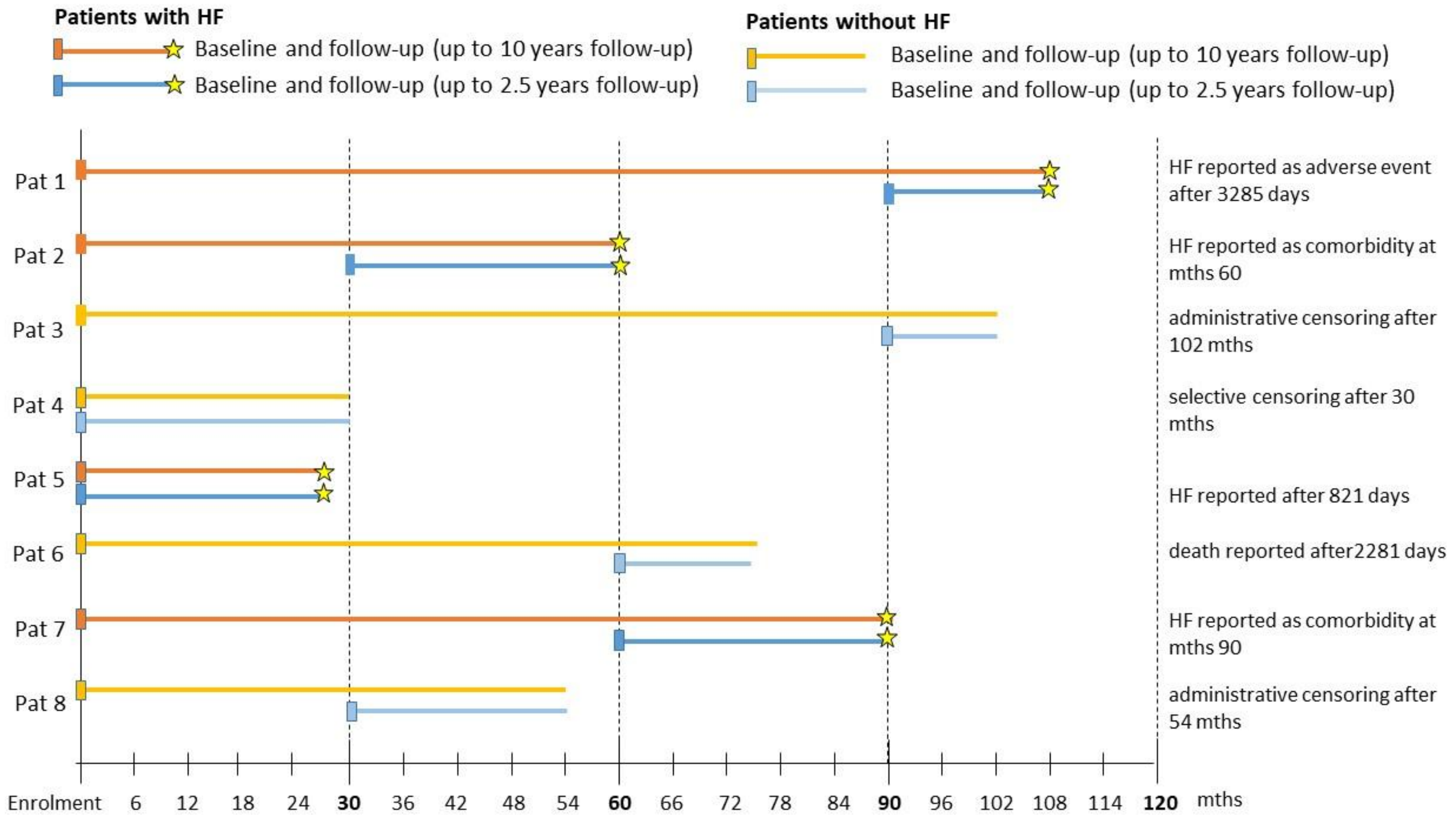

Supplemental Figure 2. Odds ratio of each predictor in a fully adjusted model (stratified by sex) for the outcome heart failure in the additional analysis (up to 2.5 years of follow-up)

Effect estimator	Men OR (95% CI)	Women OR (95% CI)	P value
**Traditional risk factors**			
Age	1.04 (1.03,1.06)	1.05 (1.04,1.06)	0.26
Hypertension	1.52 (1.07,2.17)	1.76 (1.33,2.33)	0.37
CHD	2.71 (1.95,3.75)	2.16 (1.64,2.85)	0.76
Diabetes	1.21 (0.86,1.71)	1.61 (1.25,2.08)	0.21
Hyperlipidemia	0.94 (0.65,1.37)	1.07 (0.81,1.40)	0.46
Obesity (BMI >30 kg/m^2)	1.31 (0.94,1.82)	1.08 (0.85,1.38)	0.4
Ever smoking (vs. never)	1.10 (0.76,1.57)	1.14 (0.91,1.43)	0.89
**RA-specific risk factors**			
Disease activity (DAS28-ESR)	1.24 (1.12,1.37)	1.19 (1.10,1.29)	0.44
CRP >=5 mg/l (vs. <5 mg/l)	1.26 (0.91,1.74)	1.40 (1.10,1.77)	0.88
RA disease duration	1.01 (0.99,1.03)	1.01 (1.00,1.02)	0.52
RF/ACPA positivite (vs. negative)	0.87 (0.61,1.24)	1.02 (0.79,1.33)	0.47
GC during the past six months			0.45
5-10 mg/d (vs. <5 mg/d)	1.58 (1.14,2.18)	1.25 (0.99,1.58)	
>= 10 mg/d (vs. <5 mg/d)	1.56 (0.98,2.48)	1.50 (1.01,2.22)	
NSAID and/or Cox-II-Inhibitor	1.36 (0.99,1.85)	1.01 (0.81,1.26)	0.09

Men

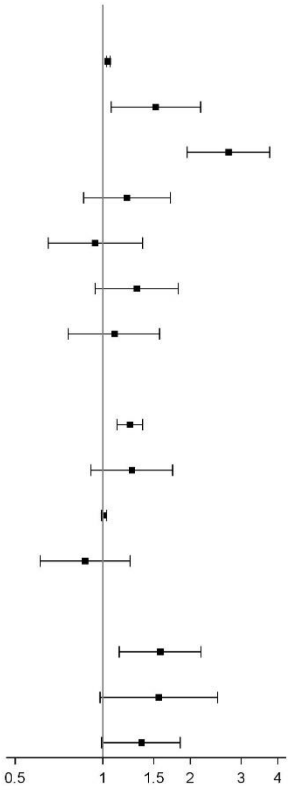

Women

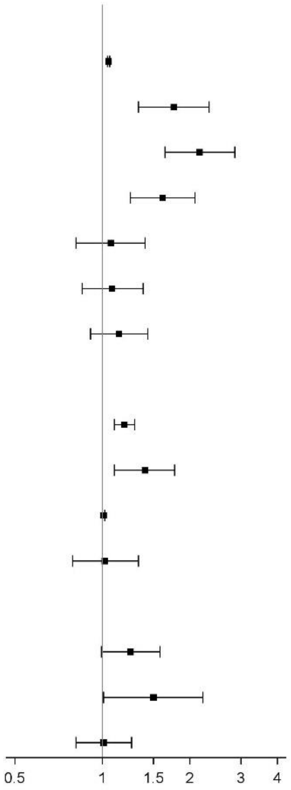

P Value: p-value of the interaction term.

All analyses are adjusted for selective drop out using inverse probability of censoring weighting.

Abbreviations: ACPA: Anti-citrullinated protein antibodies; BMI: Body mass index; CHD: Coronary heart disease; CI: Confidence interval; Cox-II-inhibitor: inhibitor of cyclooxygenase-II; CRP: C-reactive protein; GC: glucocorticoids; NSAID: Non-steroidal anti-inflammatory drugs; OR: Odds ratio; RF: Rheumatoid factor

Supplemental Figure 3. Odds ratio of each predictor in a fully adjusted model (stratified by sex) for the secondary outcome (combined heart failure or cardiac death) for A) main analysis and B) additional analysis

A) Main analysis (up to 10 years of follow-up)

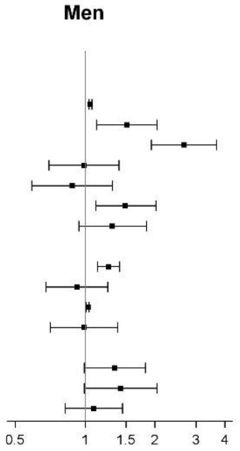

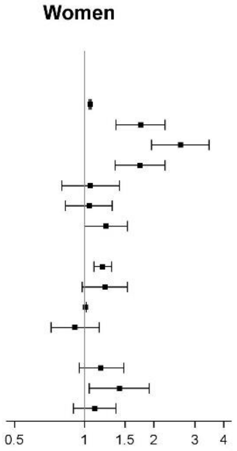

Effect estimator	Men OR (95% CI)	Women OR (95% CI)	P value
**Traditional risk factors**			
Age	1.05 (1.04, 1.07)	1.06 (1.05, 1.07)	0.21
Hypertension	1.51 (1.12, 2.04)	1.75 (1.37, 2.23)	0.26
CHD	2.67 (1.93, 3.68)	2.60 (1.95, 3.46)	0.56
Diabetes	0.99 (0.70, 1.40)	1.74 (1.36, 2.23)	0.01
Hyperlipidemia	0.88 (0.59, 1.31)	1.06 (0.80, 1.42)	0.35
Obesity (BMI >30 kg/m^2)	1.49 (1.11, 2.02)	1.05 (0.83, 1.32)	0.25
Ever smoking (vs. never)	1.31 (0.94, 1.84)	1.24 (1.00, 1.54)	0.78
**RA-specific risk factors**			
Disease activity (DAS28-ESR)	1.26 (1.13, 1.41)	1.20 (1.10, 1.31)	0.87
CRP >=5 mg/l (vs. <5 mg/l)	0.92 (0.68, 1.25)	1.23 (0.98, 1.54)	0.22
RA disease duration	1.03 (1.01, 1.04)	1.01 (1.00, 1.02)	0.24
RF/ACPA positivite (vs. negative)	0.99 (0.71, 1.38)	0.91 (0.72, 1.16)	0.71
GC during the past six months			0.76
5-10 mg/d (vs. <5 mg/d)	1.34 (0.99, 1.82)	1.18 (0.95, 1.48)	
>= 10 mg/d (vs. <5 mg/d)	1.42 (0.99, 2.04)	1.42 (1.05, 1.91)	
NSAID and/or Cox-II-Inhibitor	1.09 (0.82, 1.45)	1.11 (0.90, 1.37)	0.73

B) Additional analysis (up to 2.5 years of follow-up)

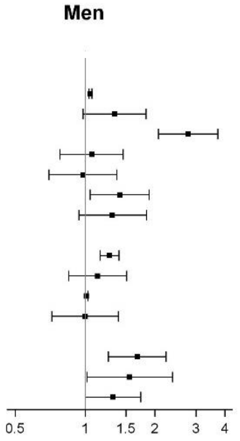

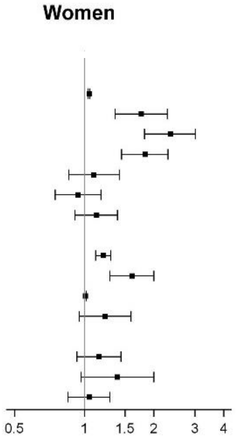

Effect estimator	Men OR (95% CI)	Women OR (95% CI)	P value
**Traditional risk factors**			
Age	1.05 (1.04, 1.07)	1.05 (1.04, 1.06)	0.19
Hypertension	1.34 (0.98, 1.83)	1.76 (1.36, 2.28)	0.12
CHD	2.78 (2.07, 3.73)	2.35 (1.82, 3.02)	0.99
Diabetes	1.07 (0.78, 1.46)	1.83 (1.45, 2.30)	0.01
Hyperlipidemia	0.98 (0.70, 1.37)	1.10 (0.86, 1.42)	0.37
Obesity (BMI >30 kg/m^2)	1.41 (1.05, 1.89)	0.94 (0.75, 1.18)	0.14
Ever smoking (vs. never)	1.31 (0.94, 1.84)	1.13 (0.91, 1.39)	0.57
**RA-specific risk factors**			
Disease activity (DAS28-ESR)	1.27 (1.16, 1.40)	1.21 (1.12, 1.30)	0.64
CRP >=5 mg/l (vs. <5 mg/l)	1.13 (0.85, 1.51)	1.61 (1.29, 2.00)	0.14
RA disease duration	1.01 (1.00, 1.03)	1.01 (1.00, 1.02)	0.78
RF/ACPA positivite (vs. negative)	1.00 (0.72, 1.39)	1.23 (0.95, 1.59)	0.39
GC during the past six months			0.14
5-10 mg/d (vs. <5 mg/d)	1.68 (1.26, 2.23)	1.16 (0.93, 1.44)	
>= 10 mg/d (vs. <5 mg/d)	1.55 (1.02, 2.38)	1.39 (0.97, 2.00)	
NSAID and/or Cox-II-Inhibitor	1.32 (1.00, 1.74)	1.05 (0.85, 1.29)	0.09

P Value: p-value of the interaction term;

All analyses are adjusted for selective drop out using inverse probability of censoring weighting;

Abbreviations: ACPA: Anti-citrullinated protein antibodies; BMI: Body mass index; CHD: Coronary heart disease; CI: Confidence interval; Cox-II-inhibitor: inhibitor of cyclooxygenase-II; CRP: C-reactive protein; GC: glucocorticoids; NSAID: Non-steroidal anti-inflammatory drugs; OR: Odds ratio; RF: Rheumatoid factor

Supplemental Table 1. Baseline characteristics stratified by sex – number of patients with missing data (n (%))

	Main analysis (up to 10 years follow-up)		Additional analysis (up to 2.5 years follow-up)	
	Men (n=4022)	Women (n=11785)	Men (n=4022)	Women (n=11785)
**Demographics and clinical information**				
Age (years)	0	0	0	0
Disease duration (years)	5 (0.1)	24 (0.2)	5 (0.1)	24 (0.2)
Rheumatoid factor and/or ACPA positive	205 (5.1)	656 (5.6)	206 (5.1)	662 (5.6)
Joint erosions	208 (5.2)	592 (5.0)	208 (5.2)	592 (5.0)
RA disease activity (DAS28-ESR)	369 (9.2)	1060 (9.0)	505 (12.6)	1434 (12.2)
Erythrocyte sedimentation rate (mm/h)	329 (8.2)	901 (7.6)	460 (11.4)	1278 (10.8)
C-reactive protein (mg/l)	387 (9.6)	1154 (9.8)	560 (13.9)	1546 (13.1)
Swollen joint count [0-28]	7 (0.2)	18 (0.2)	31 (0.8)	133 (1.1)
Tender joint count [0-28]	9 (0.2)	20 (0.2)	30 (0.7)	137 (1.2)
% of full physical function (FFbH) [0-100]	68 (1.7)	188 (1.6)	71 (1.8)	270 (2.3)
Patient reported global health [0-10]	36 (0.9)	145 (1.2)	64 (1.6)	250 (2.1)
Patient reported fatigue [0-10]	42 (1.0)	141 (1.2)	69 (1.7)	260 (2.2)
Patient reported pain [0-10]	29 (0.7)	116 (1.0)	60 (1.5)	229 (1.9)
**Previous treatments**				
No. of previous csDMARDs	0	0	0	0
No. of previous bDMARDs	0	0	0	0
No. of previous tsDMARDs	0	0	0	0
GC dosage 6 months prior to baseline	96 (2.3)	338 (2.9)	287 (7.1)	1070 (9.1)
**Start of DMARDs at enrolment**				
Tumour necrosis factor inhibitor	0	0	0	0
Interleukin-6 inhibitor	0	0	0	0
Abatacept	0	0	0	0
Rituximab	0	0	0	0
Janus kinase inhibitor	0	0	0	0
csDMARD	0	0	0	0
No DMARD	0	0	0	0
**Concomitant medication**				
b/tsDMARD combined with csDMARD	0	0	0	0
Oral GC	84 (2.1)	312 (2.6)	121 (3.0)	457 (3.9)
NSAID or Cox-II-Inhibitor	0	0	0	0
**History of comorbidities**				
N. of comorbidities	0	0	186 (4.6)	694 (5.9)
Hypertension	0	0	92 (2.2)	384 (3.3)
Coronary heart disease	0	0	159 (4.0)	662 (5.6)
Stroke / transient ischemic attack	0	0	177 (4.4)	685 (5.8)
Hyperlipidemia	0	0	157 (3.9)	617 (5.2)
Diabetes mellitus	0	0	151 (3.8)	604 (5.1)
Chronic obstructive pulmonary disease / lung fibrosis	0	0	169 (4.2)	660 (5.6)
Chronic renal disease	0	0	170 (4.2)	662 (5.6)
Cancer	0	0	158 (3.9)	651 (5.5)
Osteoporosis	0	0	149 (3.7)	546 (4.6)
Body mass index ≥30 kg/m^2	55 (1.4)	139 (1.2)	55 (1.4)	139 (1.2)
Ever smoking	31 (0.8)	130 (1.1)	47 (1.2)	216 (1.8)

Abbreviations: ACPA: Anti-citrullinated protein antibodies; bDMARD: Biologic disease-modifying anti-rheumatic drug; Cox-II inhibitor: inhibitors of cyclooxygenase-II; csDMARD: Conventional synthetic disease-modifying anti-rheumatic drug; DAS28-ESR: Disease Activity Score of 28 joints; FFbH: Hannover Functional Status Questionnaire; GC: glucocorticoid; NSAID: non-steroidal anti-inflammatory drug; tsDMARD: Targeted synthetic disease-modifying anti-rheumatic drug.

Supplemental Table 2. Baseline characteristics stratified by sex, for the additional analysis with up to 2.5 years of follow-up

	**Men (n=4022)**	**Women (n=11785)**
**Demographics and clinical information**		
Age (years)	61.1±11.8	60.4±13.1
Age <50 years	632 (15.7)	2239 (19.0)
RA disease duration (years)	10.2±8.2	12.9±9.7
RF and/or ACPA seropositive	2927 (76.7)	8503 (76.4)
Joint erosions	2111 (55.3)	5906 (52.8)
RA disease activity (DAS28-ESR)	3.6±1.6	3.7±1.5
ESR (mm/h)	22.4±21.7	21.5±19.2
ESR > 21 mm/h	1312 (36.8)	3841 (36.6)
CRP (mg/l)	11.3±19.8	8.1±13.9
CRP ≥ 5 mg/l	1609 (46.5)	4056 (39.6)
Swollen joint count [0-28]	2.6±4.2	2.5±3.9
Tender joint count [0-28]	4.1±5.8	4.2±5.8
% of full physical function (FFbH) [0-100]	73.8±22.6	66.4±24.0
Patient reported global health [0-10]	4.6±2.3	4.8±2.3
Patient reported fatigue [0-10]	4.1±2.7	4.7±2.8
Patient reported pain [0-10]	4.4±2.5	4.7±2.6
**Previous treatments**		
N. of previous csDMARDs	1.7±1.0	2.0±1.0
N. of previous bDMARDs	0.8±0.9	0.9±0.9
N. of previous tsDMARDs	0±0.2	0±0.2
GC dosage 6 months prior to baseline		
<5 mg/d	2352 (63.0)	7070 (66.0)
5 - < 10 mg/d	1041 (27.9)	2998 (28.0)
≥ 10 mg/d	342 (9.2)	647 (6.0)
**Current DMARD treatment**		
Tumour necrosis factor inhibitor	1582 (39.3)	4422 (37.5)
Interleukin-6 inhibitor	378 (9.4)	1159 (9.8)
Abatacept	220 (5.5)	622 (5.3)
Rituximab	273 (6.8)	868 (7.4)
tsDMARDs	309 (7.7)	886 (7.5)
csDMARDs	1065 (26.5)	3090 (26.2)
No DMARD	195 (4.8)	738 (6.3)
**Concomitant medication**		
b/tsDMARDs combined with csDMARD	1859 (67.3)	4645 (58.4)
Oral GC	2492 (63.9)	6865 (60.6)
NSAID or Cox-II-Inhibitor	1428 (35.5)	4767 (40.4)
**History of comorbidities**		
N. of comorbidities*	1.4±1.4	1.3±1.3
Hypertension	2060 (52.4)	5667 (49.7)
Coronary heart disease	619 (16.0)	750 (6.7)
History of stroke / transient ischemic attack	171 (4.4)	298 (2.7)
Hyperlipidemia	639 (16.5)	1476 (13.2)
Diabetes	646 (16.7)	1461 (13.1)
Chronic obstructive pulmonary disease / lung fibrosis	431 (11.2)	785 (7.1)
Chronic renal disease	292 (7.6)	836 (7.5)
Cancer	282 (7.3)	736 (6.6)
Osteoporosis	525 (13.6)	2774 (24.7)
Obesity (body mass index ≥30 kg/m^2)	1027 (25.9)	3168 (27.2)
Smoking, ever	3070 (77.2)	5868 (50.7)

*Values are numbers (percent) or means ± standard deviation; * N. of comorbidities comprises sum of: hypertension, coronary heart disease, stroke / transient ischemic attack, hyperlipidemia, diabetes, chronic obstructive pulmonary disease / lung fibrosis, chronic renal disease, cancer, osteoporosis;*

Abbreviations: ACPA: Anti-citrullinated protein antibodies; bDMARD: Biologic disease-modifying anti-rheumatic drug; Cox-II inhibitor: inhibitors of cyclooxygenase-II; CRP: C-reactive protein; csDMARD: Conventional synthetic disease-modifying anti-rheumatic drug; DAS28-ESR: Disease Activity Score in 28 joints; ESR: Erythrocyte sedimentation rate; FFbH: Hannover Functional Status Questionnaire; GC: glucocorticoid; NSAID: non-steroidal anti-inflammatory drug; RF: Rheumatoid factor; tsDMARD: Targeted synthetic disease-modifying anti-rheumatic drug

Supplemental Table 3. Association between sex and heart failure, with females used as reference, for the additional analysis with up to 2.5 years of follow-up

	Model	OR	95% CI
HF overall (ischemic & non-ischemic)	1	1.68	1.41-2.01
	2	1.52	1.25-1.85
Ischemic HF	1	2.78	2.04-3.79
	2	2.52	1.79-3.53
Non-ischemic HF	1	1.31	1.05-1.63
	2	1.33	1.05-1.88

Model 1: adjusted for age.
Model 2: adjusted for age, history of CHD (for HF altogether), hypertension, diabetes, hyperlipidemia, obesity (defined as BMI ≥30kg/m^2), ever smoking (former and current smoking), disease activity assessed by DAS28-ESR, CRP (≥5 mg/l versus <5 mg/l), RA disease duration, RF and/or ACPA seropositive, mean GC dose 6 months prior to baseline (<5mg/day, 5-10 mg/day, ≥10mg/day) and concomitant NSAID and/or COX-II-inhibitor use.

Abbreviations: ACPA: anti-citrullinated protein antibodies; BMI: body mass index; CHD: coronary heart disease; Cox-II inhibitor: inhibitors of cyclooxygenase-II; CRP: C-reactive protein; DAS28-ESR: Disease Activity Score in 28 joints; ESR: erythrocyte sedimentation rate; GC: glucocorticoids; HF: heart failure; NSAID: non-steroidal anti-inflammatory drug; RA: rheumatoid arthritis; RF: rheumatoid factor.

Supplemental Table 4. Association between sex and secondary outcome (combination of heart failure or cardiac death), with females used as reference

	Main analysis (up to 10 years of follow-up)		Additional analysis (up to 2.5 years of follow-up)	
	OR	95% CI	OR	95% CI
Model 1	1.79	1.52-2.11	1.84	1.57-2.17
Model 2	1.60	1.33-1.92	1.63	1.36-1.95

Model 1: adjusted for age.
Model 2: adjusted for age, history of CHD, hypertension, diabetes, hyperlipidemia, obesity (defined as BMI ≥30kg/m^2), ever smoking (former and current smoking), disease activity assessed by DAS28-ESR, CRP (≥5 mg/l versus <5 mg/l), RA disease duration, RF and/or ACPA seropositive, mean GC dose 6 months prior to baseline (<5mg/day, 5-10 mg/day, ≥10mg/day) and concomitant NSAID and/or COX-II-inhibitor use.

Abbreviations: ACPA: anti-citrullinated protein antibodies; BMI: body mass index; CHD: coronary heart disease; Cox-II inhibitor: inhibitors of cyclooxygenase-II; CRP: C-reactive protein; DAS28-ESR: Disease Activity Score in 28 joints; ESR: erythrocyte sedimentation rate; GC: glucocorticoids; HF: heart failure; NSAID: non-steroidal anti-inflammatory drug; RA: rheumatoid arthritis; RF: rheumatoid factor.

Supplemental Table 5. P-values for each predictor in the fully adjusted model stratified by sex (main and additional analyses for primary and secondary outcome)

Outcome	Primary outcome: heart failure				Secondary outcome: heart failure or cardiac death			
Observed time period	Main analysis (up to 10 years follow-up)		Additional analysis (up to 2.5 years follow-up)		Main analysis (up to 10 years follow-up)		Additional analysis (up to 2.5 years follow-up)	
	Men	Women	Men	Women	Men	Women	Men	Women
**Traditional risk factors**								
Age	**<0.0001**	**<0.0001**	**<0.0001**	**<0.0001**	**<0.0001**	**<0.0001**	**<0.0001**	**<0.0001**
Hypertension	**0.0211**	**<0.0001**	**0.0196**	**<0.0001**	**0.0070**	**<0.0001**	0.0650	**<0.0001**
Coronary heart disease	**<0.0001**	**<0.0001**	**<0.0001**	**<0.0001**	**<0.0001**	**<0.0001**	**<0.0001**	**<0.0001**
Diabetes	0.4445	**0.0053**	0.2693	**0.0002**	0.9616	**<0.0001**	0.6926	**<0.0001**
Hyperlipidemia	0.8299	0.7013	0.7623	0.6282	0.5390	0.6780	0.9070	0.4355
Obesity (BMI ≥30 kg/m^2)	0.0865	0.2397	0.1071	0.5309	**0.0090**	0.6936	**0.0230**	0.6062
Ever smoking (vs never smoking)	0.6277	0.1570	0.6188	0.2523	0.1145	**0.0454**	0.1121	0.2642
**RA-specific risk factors**								
RA disease activity (DAS28-ESR)	**<0.0001**	**0.0005**	**<0.0001**	**<0.0001**	**<0.0001**	**<0.0001**	**<0.0001**	**<0.0001**
CRP ≥5 mg/l (vs. <5 mg/l)	0.9542	0.5569	0.1609	**0.0052**	0.6050	0.0789	0.4094	**<0.0001**
RA duration	**0.0436**	**0.0022**	0.2772	**0.0058**	**0.0008**	**0.0042**	0.0529	**0.0491**
RF/ACPA positivity	0.3975	0.1054	0.4463	0.8609	0.9487	0.4572	0.9756	0.1085
Prior GC dosage (<5 mg/d as reference)								
5-10 mg/d	0.1992	0.1555	**0.0053**	0.0618	0.0582	0.1408	**0.0004**	0.1841
≥ 10 mg/d	**0.0482**	**0.0158**	0.0607	**0.0443**	0.0581	**0.0218**	**0.0419**	0.0749
NSAID and/or Cox-II-inhibitor	0.3834	0.2998	0.0537	0.9324	0.5380	0.3423	0.0522	0.6639

Bold: significant result (p-value <0.05).

Abbreviations: ACPA: Anti-citrullinated protein antibodies; BMI: body mass index; Cox-II inhibitor: inhibitors of cyclooxygenase-II; CRP: C-reactive protein; DAS28-ESR: Disease Activity Score of 28 joints; GC: glucocorticoid; NSAID: non-steroidal anti-inflammatory drug; RA: rheumatoid arthritis; RF: Rheumatoid factor;
